# Diffusiophoresis-Driven Stratification in Pressure-Sensitive Adhesive Films from Bimodal Waterborne Colloids

**DOI:** 10.1021/acsapm.2c02044

**Published:** 2023-01-25

**Authors:** Toby R. Palmer, Hanne M. van der Kooij, Rohani Abu Bakar, Callum D. McAleese, Mathis Duewel, Katja Greiner, Pierre Couture, Matthew K. Sharpe, Joseph L. Keddie

**Affiliations:** † Department of Physics, University of Surrey, Guildford, SurreyGU2 7XH, U.K.; ‡ Physical Chemistry and Soft Matter, Wageningen University and Research, 6708 WEWageningen, The Netherlands; § Surrey Ion Beam Centre, University of Surrey, Guildford, SurreyGU2 7XH, U.K.; ∥ Synthomer Germany GmbH, Werrastraße 10, 45768Marl, Germany

**Keywords:** stratification, pressure-sensitive adhesives, diffusiophoresis, latex, ion beam analysis, elastic recoil detection

## Abstract

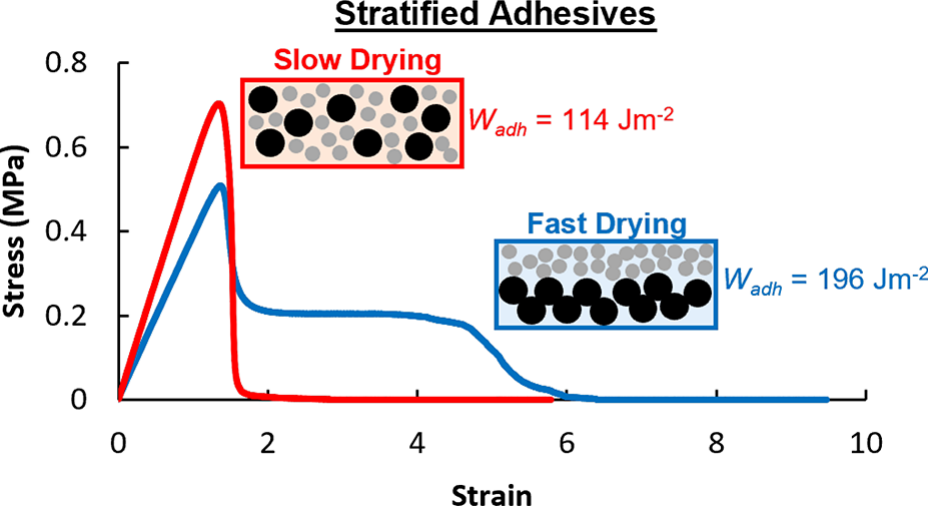

The uses of pressure-sensitive adhesives (PSAs) are wide ranging, with applications including labels, tapes, and graphics. To achieve good adhesion, a PSA must exhibit a balance of viscous and elastic properties. Previous research has found that a thin, elastic surface layer on top of a softer, dissipative layer resulted in greater tack adhesion compared with the single layers. Superior properties were achieved through a bilayer obtained via successive depositions, which consume energy and time. To achieve a multilayered structure via a single deposition process, we have stratified mixtures of waterborne colloidal polymer particles with two different sizes: large poly(acrylate) adhesive particles (ca. 660 nm in diameter) and small poly(butyl acrylate) (pBA) particles (ca. 100 nm). We used two types of pBA within the particles: either viscoelastic pBA without an added cross-linker or elastic pBA with a fully cross-linked network. Stratified surface layers of deuterium-labeled pBA particles with thicknesses of at least 1 μm were found via elastic recoil detection and qualitatively verified via the analysis of surface topography. The extent of stratification increased with the evaporation rate; films that were dried slowest exhibited no stratification. This result is consistent with a model of diffusiophoresis. When the elastic, cross-linked pBA particles were stratified at the surface, the tack adhesion properties made a transition from brittle failure to tacky. For pBA without an added cross-linker, all adhesives showed fibrillation during debonding, but the extent of fibrillation increased when the films were stratified. These results demonstrate that the PSA structure can be controlled through the processing conditions to achieve enhanced properties. This research will aid the future development of layered or graded single-deposition PSAs with designed adhesive properties.

## Introduction

Polymer films have numerous applications, including pressure-sensitive adhesives (PSAs) and protective coatings. The deposition of films from polymer colloids dispersed in water (i.e., latex) reduces the emission of volatile organic compounds (VOCs)^1^ when compared with processes using organic solvents as a carrier. However, volatile plasticizers and coalescing aids in waterborne coatings and adhesives contribute substantially to indoor air pollution.^2^ Hence, there is a continued need for materials development to achieve target properties without environmental damage. One promising strategy to eliminate VOCs is to mix waterborne colloids of different types to make a nanocomposite film. For instance, a mixture of film-forming colloids and hard fillers is often used in coatings.^3,4^ In PSA films, which are the focus of this present work, mixtures of particles with differing gel contents,^5^ molecular weight distributions,^5,6^ and sizes^6,7^ have been explored as a way to adjust the adhesive properties.

PSAs adhere to nearly any surface under the application of light pressure and have applications ranging from tapes, labels, and bandages to graphics and mechanical joints in aircraft.^8,9^ For optimal adhesive performance, PSAs require a delicate balance between viscous properties to dissipate energy during debonding and elastic properties to support stress. Hence, adhesive properties depend strongly on the molecular weight distribution, entanglement molecular weight, and cross-linked network.^10−12^

There is some evidence in the literature that a gradient (or step) in the composition of PSAs (and hence their molecular and viscoelastic properties) can be used to tailor and optimize the adhesive properties.^13−17^ One way to achieve gradient compositions is through a multilayered structure achieved via multiple depositions. The casting of multiple layers on top of one another and the lamination of layers are both technically possible, but such methods can be time-consuming, energy-intensive, and expensive for manufacturers.

Two examples of PSA bilayers in the literature are inspirational to the present research. Carelli et al.^13^ studied the tack adhesion of bilayer films composed of a more elastic layer and a more dissipative layer in each of two configurations. The use of bilayer systems allowed the surface to have different viscoelastic properties to the bulk. When using a high energy adherend surface (in their case, steel), they found that having a thin elastic layer on top of a thicker dissipative layer increased the work of adhesion by ca. 30%, when compared to the individual components on their own. The presence of an elastic surface layer also altered the debonding mechanism, transitioning it from cohesive to adhesive failure. In other work, Wang et al.^17^ likewise found that just a thin elastic surface layer applied on top of the bulk adhesive could be used to tune the tack adhesion. Specifically, they observed an increase in the work of adhesion for a bilayer containing a 3 μm surface layer of a stiffer, higher modulus adhesive on top of a 49 μm adhesive underlayer.

In the work of both Carelli et al. and Wang et al., it was found that a stiffer, more elastic-like surface layer on top of a softer, dissipative layer improved the adhesion. Contrary to this finding, other groups have shown that inverting this orientation can improve adhesion.^14−16^ Díez-Garcia et al.^16^ found that casting a liquid-like layer on top of a more solid-like layer allowed for fibrillation to occur during debonding, as was shown by the lengthened stress plateau in probe tack curves, which increased the adhesion energy.

In the prior work reviewed here, the benefit of using bilayer adhesives to tune the properties and to improve the performance of a PSA has been shown. The adhesion properties of bilayer (or multilayer) PSAs ultimately are highly sensitive to the fine balance of viscoelastic properties of each layer. To date, the fabrication of bilayer (or gradient) structures used the deposition of two (or more) separate layers. A far more attractive process is to have two or more different types of particles within the same colloidal dispersion (e.g., a mixture of viscous and elastic particles). During the evaporation of water, the particles could then separate, effectively forming a two-layered or graded structure from a single film deposition, in which the sublayers run parallel to the substrate.

The separation of particles into layers is known as stratification. There have been numerous reports of experiments and simulations of stratification in colloidal films,^18−35^ as has been outlined in review articles.^36,37^

**Figure 1:**
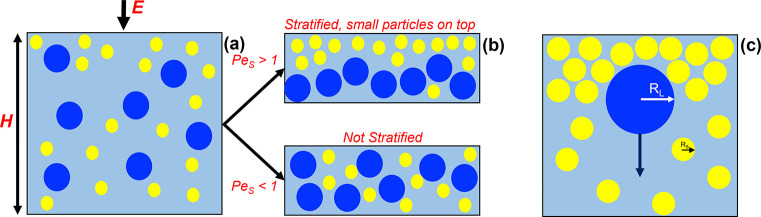
An illustration of stratification driven by diffusiophoretic motion (a) of a large (blue) particle down a gradient of smaller (yellow) particles, starting from an initially homogeneous mixture of particles (b). The final mixture can be either stratified or nonstratified (c).

The distribution of individual particles in a drying film of initial thickness, *H*, can be defined by the Peclet number, Pe. Pe describes the competition between the speed of the descending film/air interface as water evaporates at a velocity, *E*, and the rate of diffusion of the particles away from the high concentration region just below the film/air interface, typically found for dilute colloids from the Stokes–Einstein diffusion coefficient, *D*_SE_. (At higher colloids content, particle crowding affects the diffusion coefficient.) The initial state is illustrated in Figure 1a. Pe is given quantitatively as
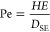
1For the case that Pe > 1, the evaporation of water is fast compared to particle diffusion, and hence the particles are trapped by the descending film/air interface, accumulating at the top surface. For fast diffusers, Pe < 1. The particles can outrun the descending interface to yield a more homogeneous distribution of particles during drying. If two differently sized particles are mixed together into a single dispersion (i.e., large (L) and small (S) particles) and have Pe_L_ > Pe_S_ > 1, the stratification of small particles on the top surface was discovered in both experimental and computational work,^20−27,33^ as is illustrated in Figure 1b.

Sear and Warren^28^ and later Sear^29^ developed a model of diffusiophoresis to describe the stratification of small particles (or polymer coils) to the top surface of larger colloidal particles. Diffusiophoresis is the motion of one species in response to the concentration gradient of another. Considering hydrodynamic effects, Sear and Warren^28^ argued that the stratification is driven by a concentration gradient of small particles that drives the motion of larger colloids downward (Figure 1c) to reduce the interfacial free energy at the interface between the large particles and the colloidal dispersion. Sear and Warren found qualitative agreement with a model from Zhou et al.,^33^ but by including the solvent backflow, they found that previous models overestimated the downward velocity of large particles, suggesting stratification would be less pronounced than expected.

Sear’s model^29^ considered the impact of a jammed layer of small particles below the descending film/air interface. For sufficiently high volume fractions of small particles, ϕ_S_, the small particles form a jammed, solid-like structure that moves downward during water evaporation. For stratification to be observed, the downward diffusiophoretic velocity of the large particles must be greater than the velocity of the jammed layer, such that they can escape the descending film/air interface. If the initial volume fraction of small particles is too high, stratification cannot occur. It has also been shown that stratification is suppressed when the total volume fraction of particles is increased to higher values.^25^ By assuming the volume fraction of packed particles in the jammed layer, ϕ_jam_, to be 0.64 (the random packing fraction), an approximated upper limit for the volume fraction of small particles if stratification is to occur was found to be ϕ_S_ = 0.2. Above this limit, the downward diffusiophoretic velocity of large particles is too slow, and they too will become trapped at the film/air interface. In the works of Sear and Warren and Sear, particle interactions are neglected, and it is assumed that *R*_L_ ≫ *R*_S_. The boundary condition between stratified and homogeneous regimes is given by Sear and Warren^28^ as
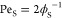
2for which Sear proposed the upper limit due to jamming as
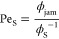
3Experimental tests of the Sear and Warren and Sear models are available in the literature,^24,30,31^ showing some agreement with the models.

More recently, Rees-Zimmerman and Routh^27^ developed a diffusion–diffusiophoresis model that accounts for the diffusion of small and large particles, diffusiophoresis effects, and the incorporation of particle-interaction terms in a drying bimodal colloidal film. They showed that to achieve small-on-top stratification arising from a downward flux of the larger particles, diffusiophoresis must be included in the model. Without it, large particles accumulate at the top surface. They suggested that diffusiophoresis must be combined with cross-interaction effects (as used by Zhou et al.) to achieve stratification. In the present work, the map from the Sear and Warren model is used because it allows a straightforward comparison to experimental data.

Typically, the intention of stratifying colloidal films is to adjust the properties of the surface compared to the bulk material. To this end, there have been several experimental studies showing how stratification can be applied to tune properties. Examples of properties include surface wetting,^30^ abrasion resistance,^38^ antibacterial properties,^39^ blocking resistance,^40^ and nanopigment^41^ and metal nanoparticle^42^ distribution. Despite these numerous examples, there is no prior work purposefully applying colloidal stratification mechanisms to optimize adhesive properties.

In this work, we studied the stratification in waterborne PSAs containing a mixture of small poly(butyl acrylate) (pBA) particles with larger, adhesive particles to investigate whether a gradient in properties can be achieved through a single film deposition and how the resulting structure influences the adhesive properties. Two types of mixtures were prepared, containing pBA particles either having no external covalent cross-linker or else having an added cross-linker to achieve extensive cross-linking of the intraparticle polymer chains. Thus, we compare the effects of viscoelastic particles and fully elastic particles. We used ion beam analysis to establish the distribution of small particles near the surface, thereby enabling some quantification of stratification. Atomic force microscopy provided complementary information about the surface morphologies. This research aims to correlate the macroscopic tack adhesion properties with the microscopic stratified structure.

## Results and Discussion

### Characterization of Components

The characteristics of the components used to prepare colloidal mixtures are presented in Table 1. Data include the molecular weight, *M*_w_, polydispersity (*Đ*), and *Z*-average particle radius, *R*, along with the corresponding polydispersity index (PDI). One type of pBA particle contained no added cross-linker (hereafter called pBA_0_). The other type of pBA particle contained 25 mol % ethylene glycol dimethacrylate (EGDMA) as a cross-linker (called pBA_25_) Two mixtures were prepared: (1) a standard acrylic PSA copolymer (called PSA2) with pBA_25_ and (2) PSA2 with pBA_0_. The PSA2 and pBA dispersions (both with a solids contents of 20 wt %) were mixed in a volume ratio of 3:1, yielding volume fractions for the small and large particles of ϕ_S_ = 0.05 and ϕ_L_ = 0.15, respectively. The total volume fraction, ϕ_total_, was 0.20.

**Table 1:** Summary of the Characteristics of Large and Small Polymer Particles

sample code	cross-linker conc (mol %)	*M*_w_(g/mol)	*Đ*	gel content (wt %)	*T*_g_ (°C)	*R* (nm)	PDI
pBA_0_	0	398000^a^	1.93	85	–46.4	60	0.06
pBA_25_	25	–b	–	100^c^	18.1	51	0.14
PSA2	n/a	259000	2.3	29	–40.0	333	1.08

a This value was obtained from the sol component in similar particles as reported by Palmer et al.,^43^ which had a gel content of 63 wt %.

b Because the gel content is 100%, the molecular weight of pBA_25_ could not be measured.

c As reported by van der Kooij et al.,^44^ the gel content within pBA_25_ is 100%.

**Figure 2:**
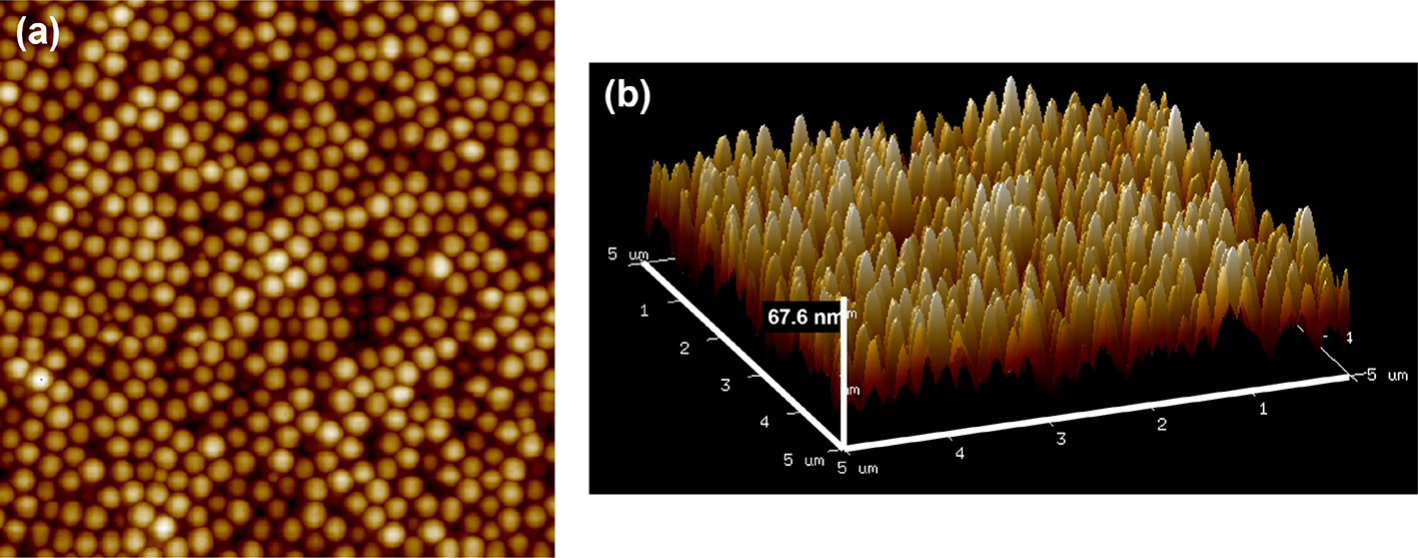
(a) AFM height image of a film made from pBA_25_ and (b) 3D side view of the same data, with an average peak-to-valley height of 49 ± 3 nm. Images are 5 μm × 5 μm.

The weight-average molecular weight for the sol components of pBA_0_ is far above the entanglement molecular weight of *M*_e_ = 25000 g/mol^45^ for pBA, which means that there will be some viscoelasticity from the entangled polymer chains. PSA2 likewise has a high molecular weight, *M*_w_ = 259000, and a low glass transition temperature, *T*_g_, that will impart viscoelasticity at room temperature. Because of the extensive cross-linking of the intraparticle polymer chains in pBA_25_, resulting in 100% gel, the particles can be thought of as fully elastic, nondeforming spheres. In Figure 2 an atomic force microscopy height image of the surface of a brittle pBA_25_ film is shown. Particles have undergone minimal deformation or coalescence, showing random packing with some limited hexagonally packed structures. Because of the brittleness of the material, it is not possible to conduct adhesion or rheological measurements on the pBA_25_ material.

**Figure 3:**
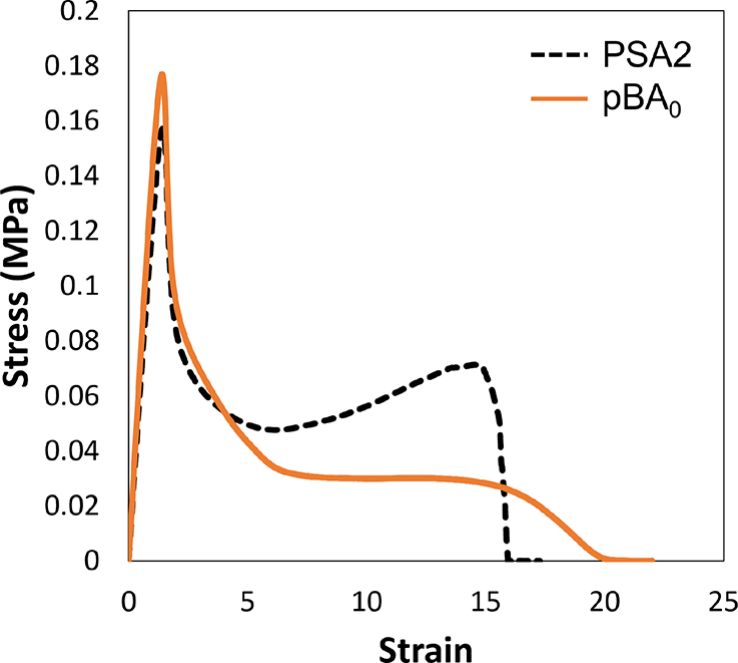
Comparative probe tack curves for two different adhesives: PSA2 and pBA_0_.

To characterize the adhesion properties of PSA2 and pBA_0_, probe tack analysis was used. During a tack test, a spherical probe is brought into contact with an adhesive film and then retracted at a constant speed. The force required to withdraw the probe from the film is obtained as a function of distance and used to produce a stress–strain curve. Presented in Figure 3 are stress–strain curves for PSA2 and pBA_0_. Both samples have a similar maximum stress and initial slope, suggesting similar wetting characteristics and elastic moduli. The fibrillation behaviors are significantly different for PSA2 and pBA_0_. There is a lower fibrillation plateau for pBA_0_ that remains steady with increasing strain, in contrast to the obvious increase in stress with strain for PSA2, which is known as strain hardening.^46,47^ With strain hardening, a greater force is required to strain the fibrils when they are stretched farther. Strain hardening is common in optimized adhesives that include some light chain cross-linking to impart rubber elasticity.^11^

From the analysis of the area under the stress–strain curve, the work of adhesion, *W*_adh_, for PSA2 is 146 ± 9 J m^–2^, which is greater than 119 ± 5 J m^–2^ that was found for pBA_0_. As is expected for a standard adhesive, PSA2 has the superior properties. Its strain hardening is consistent with the observed gel content, indicative of some cross-linking.

To explore further the linear viscoelastic properties of PSA2, pBA_0_, and the mixtures, rheometry was performed using a frequency (*f*) sweep at 25 °C, over frequencies comparable to the conditions of the tack tests. Full data showing *G*′ and *G*″ are presented in Figure S1 of the Supporting Information. For the correlation of results, it is best to study the viscoelasticity at the frequency corresponding to an initial strain rate of the probe tack test: 30 s^–1^. Selected rheological measurements at *f* = 30 Hz are presented in Table 2, with data for the mixtures that will be discussed later in the results. There are also data at *f* = 1 Hz, corresponding to the time scale for the bonding of the probe to the PSA. The rheology of PSA2 and pBA_0_ showed viscoelasticity that is attributed to entangled, high-*M*_w_ chains. Following a Soxhlet extraction, both types of particles were found to have a high-*M*_w_ sol component within a gel network, which will dissipate energy upon deformation.

**Table 2:** Selected Rheological Properties for the Components and Mixtures at Frequencies of 30 and 1 Hz

	*f* (Hz)	PSA2	pBA_0_	PSA2 + pBA_0_	PSA2 + pBA_25_
*G*′ (MPa)	30	0.38	0.32	0.42	3.5
tan(δ)/*G*′ (×10^–6^ Pa^–1^)	30	1.6	1.2	1.4	0.12
*G*′ (MPa)	1	0.1	0.2	0.1	1.5
tan(δ)/*G*′ (×10^–6^ Pa^–1^)	1	6	1.8	4.3	0.3

At the frequency of the probe tack tests, the storage modulus, *G*′, is slightly larger for PSA2 than for pBA_0_, which is expected because pBA_0_ is poly(butyl acrylate) without any optimization. The difference in *G*′ is not however significant, which is indicated by the agreement in the initial slopes of stress versus strain in Figure 3.

The Dahlquist criterion^48^ for tack adhesion (ca. 0.1 MPa) provides a rough upper limit of *G*′ to achieve good wetting of a surface by an adhesive. At the bonding frequency of the probe tack tests, 1 Hz, *G*′ values for PSA2, pBA_0_, and their mixture, PSA2 + pBA_0_ are comparable to the criterion upper limit. This explains the good tack adhesion demonstrated for PSA2 and pBA_0_ in Figure 3 and also indicates good wetting and adhesion for the PSA2 + pBA_0_ mixture. For the PSA2 + pBA_25_ mixture, *G*′ is well above the criterion, suggesting this material will not wet the probe sufficiently to achieve good adhesion.

Soxhlet extractions found that the gel content is 29 wt % for PSA2 films and 85 wt % for pBA_0_. Although no ethylene glycol dimethacrylate cross-linker was added during the synthesis of the pBA_0_ particles, a high gel fraction was found via the Soxhlet extraction. There are two explanations for this unexpected result. One, it is possible that intramolecular chain transfer occurred during the reaction (so-called “backbiting”), in which a midchain radical is formed.^49,50^ This reaction produces branching and occasional covalent cross-links between chains. Two, entanglements of long pBA chains will create physical entanglements. During the Soxhlet extraction, free polymer chains must reptate through the swollen gel network and entangled chains in the samples (approximately 1 mm thick). Any branched chains will diffuse more slowly than linear chains.

The Soxhlet extraction was run for 24 h (8.6 × 10^4^ s), but the time scales for swelling and mutual diffusion of the solvent and polymer chains in the network could be even longer, considering typical diffusion coefficients for polyacrylates^51^ on the order of 1 × 10^–14^ cm^2^/s for comparable molecular weights and temperatures. Thus, it seems fair to conclude that some non-cross-linked (possibly branched) chains in the pBA_0_ films will remain entangled during the Soxhlet extraction and contribute to the measured gel content. In comparison, there is dense covalent cross-linking in the pBA_25_ particles (with added cross-linker), resulting in 100% gel content with a low molecular weight between cross-links leading to a high elastic modulus.

Deplace et al.^52^ proposed that the easily measurable quantity, tan(δ)/*G*′, can be used as a predictor of the extent of fibrillation. Fibrillation requires a sufficiently high dissipative component represented by tan(δ). Their research found that significant fibrillation occurs only above a tan(δ)/*G*′ = *G*″/*G*′ value of 5 × 10^–6^ Pa^–1^.^52^ Other work has found fibrillation at values greater than 3 × 10^–6^ Pa^–1^.^53^ It is important to note that there is no universally accepted value of tan(δ)/*G*′ that represents a transition to a well fibrillating material. Values should be used to compare samples relative to a given set, under consistent experimental conditions. The value of tan(δ)/*G*′ for PSA2 is greater than pBA_0_, which explains why its fibrillation is greater than for pBA_0_, as was already shown in the probe tack results in Figure 3. There is significant strain hardening of PSA2, with a plateau stress that is much larger than found for pBA_0_. Both films begin to fail at a strain of 15, with much cleaner probe detachment (abrupt drop) for PSA2 than for pBA_0_. Overall, the fibrillation (and general adhesive performance) of PSA2 is better than for pBA_0_.

### Diffusiophoretic Speed

The downward diffusiophoretic speed, *U*, of the large particles is the speed with which they move away from the film/air interface, due to the concentration gradient of small particles. *U* can be estimated using eq 4, from Sear and Warren^28^
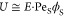
4where the parameters have each been previously defined.

The values for *U* calculated using eq 4 are presented in Table 2 to give an indication of whether downward diffusiophoresis of large particles is likely to outpace the interface motion from the evaporation of water. For *U* > *E*, large particles can escape the descending film interface, leading to the stratification of small particles on the top surface. If stratification is expected based on the relative values of *E* and *U*, then “yes” is written in the final column. Otherwise, “no” is written.

To produce samples with a range of Pe_S_, film formation conditions were changed, as listed in Table 4.

From the values presented in Table 3, the expectation of whether stratification will occur is inferred. Stratification is more likely to occur when Pe_S_ has a higher value, which is consistent with prior experimental work.^30,36^ For Pe_S_ = 19, *E* ≈ *U*, and it is a borderline case for which some weak stratification could occur.

**Table 3:** Evaporation Rates and Diffusiophoretic Speeds for the Two Mixture Types

Pe_S_	*E*(nm/s)	*U*(nm/s)	small component	stratification?
95	384	1824	pBA_0_	yes
80	384	1542	pBA_25_	yes
22	80	88	pBA_0_	yes
19	80	76	pBA_25_	borderline
3	10	2	pBA_0_	no
2	10	1	pBA_25_	no

### Stratification of Cross-Linked, Elastic Particles (pBA_25_)

In the first round of experiments, a mixture of pBA_25_ and PSA2 particles was used. In this mixture, the small particles have fully cross-linked intraparticle polymer chains, and can be thought of as elastic.

#### Surface Morphology

**Figure 4:**
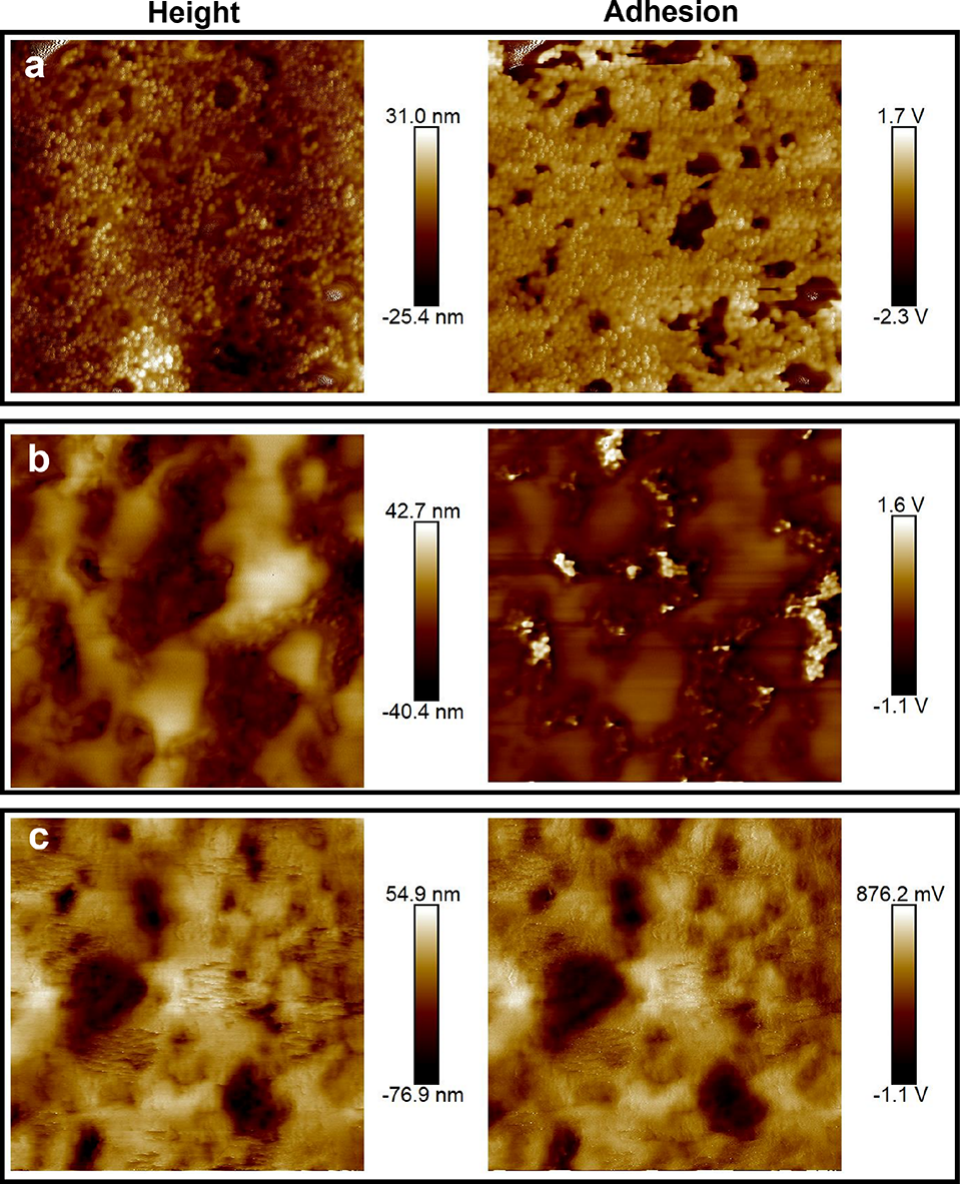
Atomic force microscopy images (height on the left; adhesion maps on the right) for films made from a pBA_25_ + PSA2 mixture under conditions for (a) Pe_S_ = 80, (b) Pe_S_ = 19, and (c) Pe_S_ = 2. All image sizes are 5 μm × 5 μm.

AFM height and adhesion images of the top surface of films formed under conditions of three different Pe_S_ are shown in Figure 4. Height images show surface topography, whereas adhesion maps show a signal related to the relative tip/sample detachment force. The sample with Pe_S_ = 80 (high evaporation rate) shows almost complete surface coverage of the small particles. Image analysis of the small particles provides an approximate particle radius, *R*, of 50 nm, which is consistent with dynamic light scattering measurements of pBA_25_ particles. For Pe_S_ = 19, there is some enrichment of small particles at the surface, but without the total coverage seen for the Pe_S_ = 80 sample. There are also regions where the small particles are covered by a smooth layer of coalesced particles, likely to be PSA2. Finally, the film formed with Pe_S_ = 2 shows a mostly smooth surface in AFM, with no small particles visible. This structure suggests a depletion of small particles at the top surface and good coalescence of the softer PSA2 particles at the surface. The images point to small particle stratification for samples with Pe_S_ = 80 and 19, but not for Pe_S_ = 2.

AFM only provides information about the surface structure of films and is subject to identification of the phases. Elastic recoil detection (ERD), which is a type of ion beam analysis, was used to obtain quantitative depth profiles of the small pBA_25_ particles labeled with deuterium.^54,55^

#### ERD Depth Profiles

ERD was conducted to determine the hydrogen and deuterium distribution in the top few micrometers of the films. The small pBA_25_ components are deuterium-labeled, allowing them to be distinguished from the large component that was free of deuterium. In ERD, incident ^4^He^+^ ions forward recoil hydrogen and deuterium (D) atoms upon collision. The energy of the atoms upon detection is used to determine the mass and depth of the forward recoiled atom into the surface. Because of its greater mass, D is forward recoiled from the surface at a higher energy than H. The counts of the higher energy edge of the peaks are a measure of the concentration of each element at the top surface of the film. As ^4^He^+^ ions penetrate farther into the film and recoiled H and D travel through the film on their exit, energy is lost. Therefore, the energies of forward-recoiled H and D are reduced, leading to a spectrum of energies for each element. These spectra are used to extract a depth profile of the H and D concentrations. In our experiments, the maximum depth probed was 2 μm, out of an expected dry film thickness of 200 μm.

**Figure 5:**
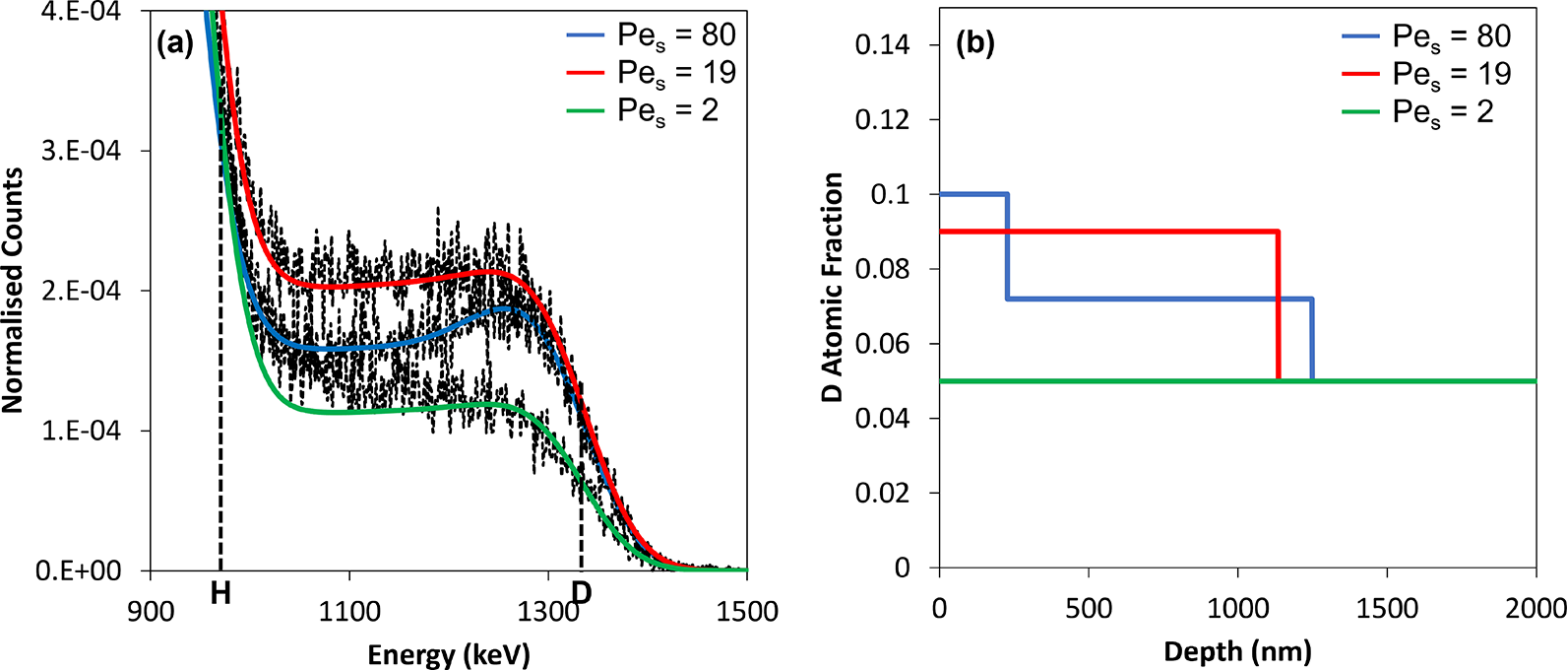
(a) Normalized ERD spectra (dashed line) and their corresponding fits (solid, colored lines) for the mixture of PSA2 with pBA_25_ for three different values of Pe_S_. (b) Depth profiles showing the atomic fraction of D as a function of the distance from the surface, as was obtained from the data in (a) to a maximum depth of ca. 2 μm.

In Figure 5 we present normalized energy spectra and the corresponding fits for the three samples. The counts of recoiled ions were normalized by the total counts below an energy of ca. 600 keV (corresponding to forward scattered He) to account for small differences in the charge collection and to allow for a more suitable visual comparison of the spectra. The full spectra of the original data, without normalization, are presented in Figure S2. Figure 5 also presents the D depth profiles that have been found through data analysis.

Simply by looking at the energy spectra in Figure 5a for the three samples analyzed with ERD, a difference in the deuterium distributions can be seen. The relative amount of deuterium at the surface (at the front edge of the D peaks, around 1300 keV) increases as Pe_S_ is increased. The counts decrease at the lower energies, which means that the D fraction is lower beneath the surface layer. A greater D fraction at the surface is explained by more of the small, cross-linked particles (pBA_25_) being stratified onto the top surface.

In the D depth profiles in Figure 5b, for the sample with Pe_S_ = 80, there is a higher D concentration at the surface, with a two-step profile that decreases to the bulk concentration after ca. 1 μm. The film with Pe_S_ = 19 likewise shows enrichment by the small deuterated particles. Both films have an enriched concentration of deuterium (and hence pBA_25_ small particles) to depths of ca. 1 μm from the surface, which is consistent with the expectation of the diffusiophoresis model in Table 3. For the sample with Pe_S_ = 2, in contrast, the D concentration is uniform with depth. A single slab representing the random mixture is used to fit the data. A slight enrichment of the hydrogen concentration—from 48.4 to 55 wt %—was required to fit the spectra. This brings the concentration of hydrogen in this simulation close to the concentration in PSA2, indicating that the film surface could be enriched in large PSA2 particles. The deuterium concentration for this sample is constant for the entire probed depth, indicating no stratification of the small particles.

These depth profiles are consistent with the AFM image analysis. The stratification of small particles has been controlled simply by changing the evaporation rate of water during the film formation. The end-user can control the stratification, depending on the desired adhesive properties, which are presented in the next subsection.

#### Probe Tack Adhesion

**Figure 6:**
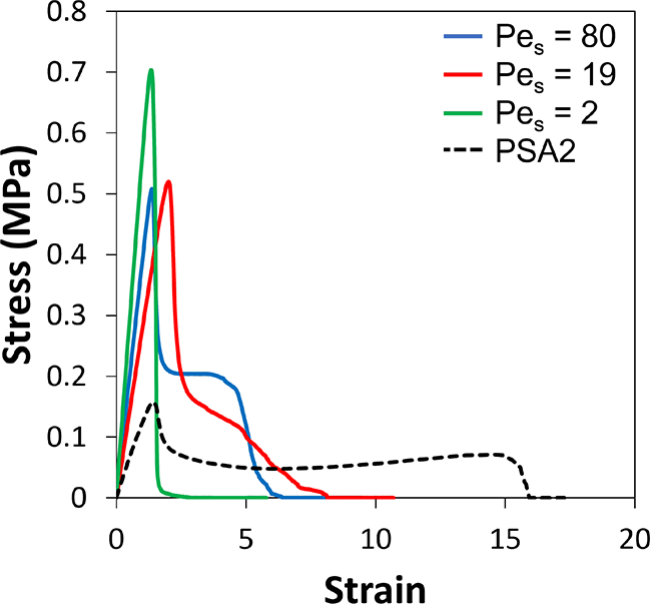
Representative probe tack curves for PSAs made from mixtures of pBA_25_ and PSA2 at three different Pe_S_ in comparison to the pure PSA2.

According to prior work on bilayers, a thin, elastic surface layer is expected to increase tack adhesion.^13,17^ Probe tack curves for PSA2 + pBA_25_ mixtures with three values of Pe_S_ are presented in Figure 6 and compared to the original PSA2. Significant differences in the curves are visible as a function of Pe_S_. In all cases, the fibrillation plateau do not extend as far for the PSA2 sample. For pBA_25_ with Pe_S_ = 80, the initial slope is higher than PSA2, which is explained by a higher modulus arising from the enrichment of elastic particles. There is a remarkably high value of the plateau stress, σ_plateau_, compared to PSA2, which means that a greater stress is needed to extend the fibrils. For Pe_S_ = 19, there is similarly a higher initial slope (suggesting a higher elastic modulus), but the fibrillation plateau is not as well-defined as for Pe_S_ = 80. The fibrils lack some of the extensibility of the higher Pe_S_ mixture, although the plateau stress is initially high. For the sample with Pe_S_ = 2, brittle failure is seen, with no fibrillation plateau, but with a higher initial slope and maximum stress.

**Figure 7:**
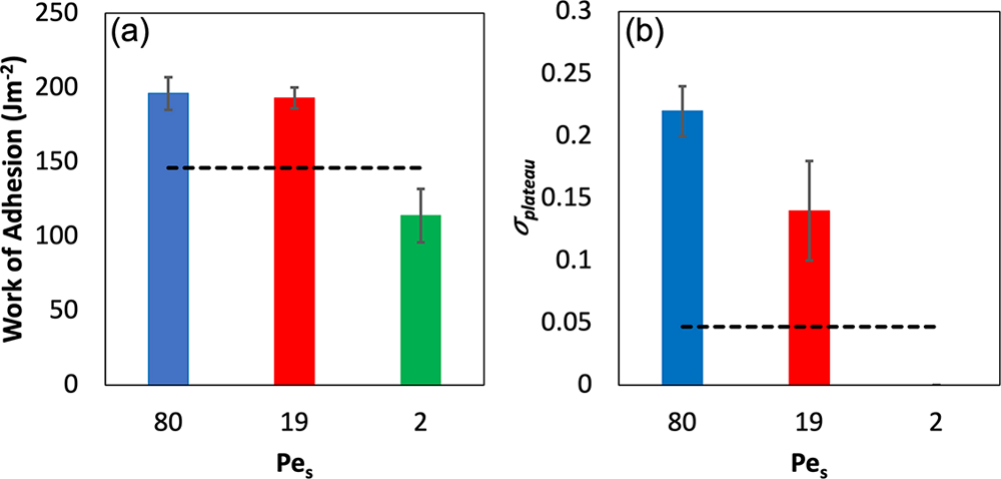
Analysis of the probe tack data for the pBA_25_ + PSA2 mixture. (a) Adhesion energy for the three samples, as a function of Pe_S_. (b) Stress of the fibrillation plateau as a function of Pe_S_. Properties of films made from large PSA2 particles only are represented by the black dashed line, for comparison. Error bars represent the standard deviation associated with five repeat measurements.

The work of adhesion, *W*_adh_, is a measure of the total amount of energy (per unit area) required to debond the probe from the adhesive surface. The plateau stress, σ_plateau_, indicates the average stress required to extend the fibrils. Both of these properties are presented in Figure 7. Both properties increase in value as the Pe_S_ is increased. The samples that have stratified (Pe_S_ = 19 and 80) have values that are greater than found for PSA2 (shown with the dashed line.) The sample that has not stratified (Pe_S_ = 2) has the lowest value of *W*_adh_ and no measurable σ_plateau_.

Insight into these results can be provided by the rheological analysis of a PSA2 + pBA_25_ mixture. To achieve a random mixture throughout the bulk material for rheology, the sample was formed by drying slowly, such that the two populations of particles were randomly mixed in the bulk material. In this mixture, tan(δ)/*G*′ = 1.2 × 10^–7^ Pa^–1^, which is a factor of 10 less than for PSA2 on its own. The implication is that with a random distribution of pBA_25_ particles in the Pe_S_ = 2 adhesive, fibrillation is inhibited because the bulk of the film does not have a sufficiently strong viscous component. *G*′ for the random mixture, meanwhile, is a factor of 10 greater than for PSA2. This is expected for a composite with the addition of a component with a higher elastic modulus.^3,4^ The value of *G*′ for the PSA2 + pBA_25_ mixture exceeds the Dahlquist criterion for tack adhesion.^48^ This fact explains why brittle adhesive failure is observed when Pe_S_ = 2. According to the ERD analysis of the Pe_S_ = 2 sample, the surface composition is similar to the PSA2 adhesive, which suggests that the elastic pBA_25_ particles are distributed throughout the bulk of the PSA film. However, with a high Pe, when there is an elastic layer stratified at the surface, the bulk of the film is less enriched by the elastic particles, and the adhesion properties have an increased value. This result is consistent with previous research^13,17^ that showed the benefits of a thin elastic layer on the surface of PSAs.

The pBA_25_ particles cannot be significantly extended due to the extensive polymer chain cross-linking, and so the film fails much sooner than does PSA2, which is designed to allow for extension during fibrillation. The increased modulus (at least in the surface region) for the stratified sample can be seen by the gradient of the initial slope in Figure 6. The application of the adhesive will dictate the trade-off between having a high plateau stress and long extension during fibrillation.

### Stratification of Viscoelastic Particles (pBA_0_)

Next, we will present data from a mixture using small pBA_0_ particles, which are viscoelastic, because no cross-linker has been added. For the pBA_0_ + PSA2 mixtures, AFM imaging shows that particles readily coalesce to form a smooth cohesive film surface, with no visible particle boundaries. This renders AFM a less effective technique, as distinguishing large from small particles is not practical. Surface morphologies showed no noteworthy differences between samples film formed at different value of Pe_S_.

#### ERD Depth Profiles

**Figure 8:**
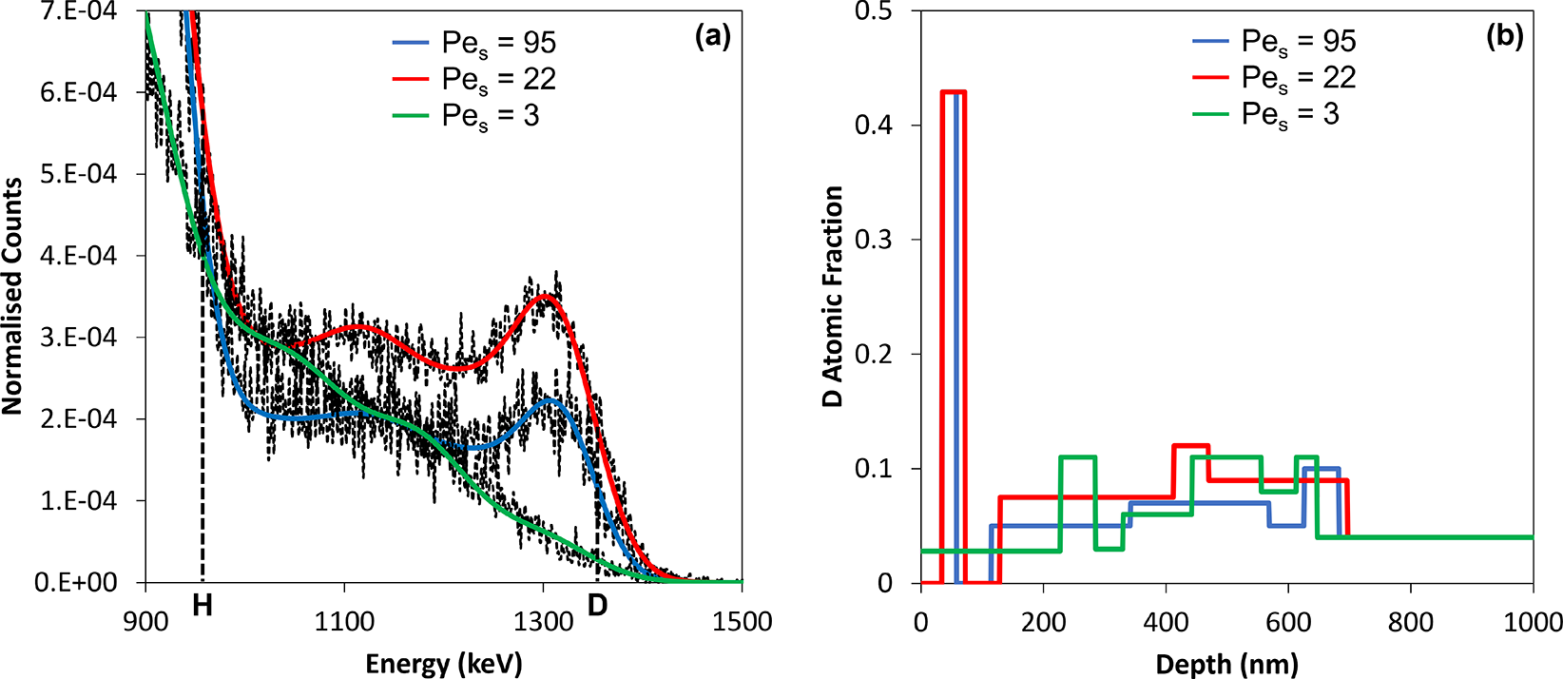
Normalized ERD data analyzed with SIMNRA. (a) ERD spectra (dashed lines) and their corresponding fits (solid, colored lines) for the pBA_0_ + PSA2 mixture for three different values of Pe_S_. (b) Depth profiles showing the concentration of deuterium through the film, obtained from the data in (a), to a maximum depth of 1 μm.

The normalized ERD data and the corresponding fits presented in Figure 8a show differences in the deuterium distribution as the values of Pe_S_ are varied. The same normalization procedure as for previous samples was used. Full spectra of the raw data, without normalization, are presented in Figure S2. Starting with Pe_S_ = 3, there are relatively few D counts at the energy corresponding to the surface. A steadily increasing number of counts is seen with decreasing energy, suggesting that D increases with depth. Indeed, in Figure 8b, the fitted D depth profile fluctuates but remains consistently low up to depths of 1000 nm. No surface enrichment of deuterium, and by extension the small pBA_0_ particles, is found, which is consistent with the diffusiophoresis model in Table 3.

The other two samples, Pe_S_ = 22 and 95, have higher D counts at the energy for the surface recoils. Qualitatively, these represent an enrichment of deuterium near the top surface of the film, with a lower amount deeper into the film. The fitted depth profiles (Figure 8b) show that both samples have a thin layer composed of pure deuterated pBA, corresponding to a D mole fraction of 0.43. For Pe_S_ = 95, this layer is 23 nm thick, and for Pe_S_ = 22 it is 37 nm thick. Below this layer, the D mole fraction drops to 0.05. The layers enriched in deuterated pBA_0_ are slightly subsurface, below a layer of pure, nondeuterated PSA2, which yields a three-layered structure. First, there is a layer of the larger component (PSA2), followed by a layer of the small particles (pBA_0_), and then finally a random mixture of large and small particles. This result has been observed elsewhere^34,56^ and is attributed to a layer of large particles becoming initially trapped at the air/film interface, with small-on-top stratification then occurring below this first layer.

As shown in Figure 8b, the layer thicknesses in the ERD best-fit models are as thin as 40 nm, which is less than the size of an individual pBA_0_ particle whose radius is 60 nm. For this mixture, obtaining such a distinct layer of the small component is not realistic, given the ease with which pBA_0_ particles will deform and coalesce. Both large and small components in this mixture will have an interfacial width, which also explains the lack of clearly distinguishable pBA_0_ layers in the ERD depth profiles. Crucially, the ERD show that the extent of stratification increases as the Pe_S_ is increased.

ERD is only able to probe to a depth of 1 or 2 μm from the surface, but we have successfully resolved sublayers on the order of 40 nm. By contrast, small-angle X-ray scattering (SAXS) using a microbeam has been used to obtain concentration profiles through stratified films up to several 100 μm^56,57^ thick, although it does not offer the high resolution of ERD (on the order of tens of nanometers^58−60^). Similarly, Raman mapping^31^ provides depth profiles over large distances but without the high resolution obtained with ERD.

#### Probe Tack Adhesion

**Figure 9:**
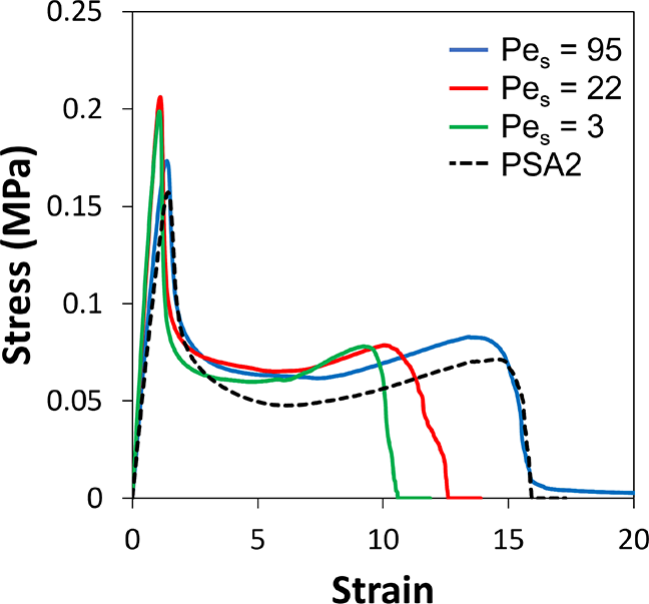
Probe tack curves for PSAs made from mixtures of pBA_0_ and PSA2, upon film formation with three different values of Pe_S_. A curve for PSA2 is shown for comparison.

Probe tack curves for the mixture film formed with three different Pe_S_ are presented in Figure 9, in comparison to PSA2. In all cases, a reasonable extension of the fibrils is obtained as is revealed by the long plateau length. The values of σ_plateau_ are consistently higher for the mixture than for PSA2, regardless of the value of Pe_S_. As such, this parameter shows an effect of a composite but does not indicate an effect of stratification itself. The addition of the pBA_0_ particles with no added cross-linker have a reinforcing effect on the fibrils, but the effect on σ_plateau_ is less than found when elastic, cross-linked particles (pBA_25_) were added.

**Figure 10:**
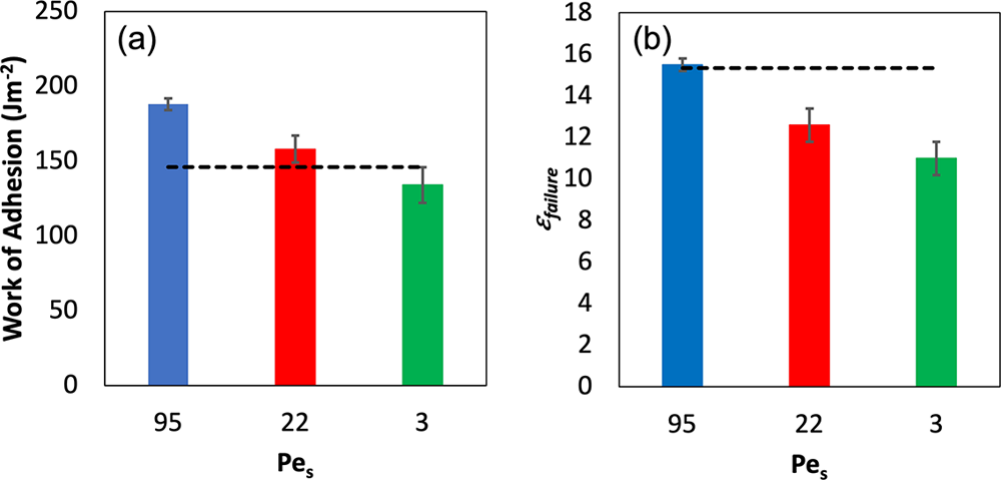
Analysis of the probe tack data for the pBA_0_ + PSA2 mixture. (a) Work of adhesion for the three samples with differing Pe_S_ shown in the legend. (b) Strain at failure for three different Pe_S_ used in film formation. In both graphs, data from a PSA2 film are represented by the black dashed lines for comparison. Error bars represent the standard deviation associated with five replicate measurements.

The strain at failure, ϵ_failure_, is found to increase with increasing Pe_S_, as is shown in Figure 10b. Drawing on the rheological data presented in Table 2, it is not expected that a random mixture of PSA2 and pBA_0_ will fibrillate farther than the PSA2 on its own. The value of tan(δ)/*G*′ of 1.4 × 10^–6^ for the mixture falls between the values for the components, PSA2 and pBA_0_. It is therefore understandable that the mixture does not fibrillate more than PSA2. Fibril extension is increased with increasing values of Pe_S_, for which stratification has been established. There is some benefit from the surface enrichment of the mixture with more liquid-like pBA_0_ particles, but ϵ_failure_ does not exceed what is found for PSA2.

The greater σ_plateau_ for the PSA mixtures leads to a greater value for *W*_adh_ when Pe_S_ = 95 and 22 (but not when Pe_S_ = 3) in comparison to PSA2. Considering the ERD depth profiles, it can be inferred that stratification of the viscoleastic pBA_0_ particles, as was found for Pe_S_ = 95 and 22, translates to a greater *W*_adh_, as shown in Figure 9a. In addition to the effects of a surface layer, there is likely an effect of the viscoelastic properties of the composite, as shown in Table 2. The *G*′ for the mixture (0.42 MPa) is larger than for the components on their own, which could lead to a greater stress for fibril extension.

Comparison of the ERD data reveals that the stratified layer is much thicker for the highly cross-linked pBA_25_ particles than for the pBA_0_ particles presented in this section. Differences in adhesion between the mixed components could have an effect on the stratification. pBA_0_ particles are more prone to deformation than pBA_25_, meaning they will experience flat-faced contact with the deforming PSA2 particles. There is likely to be some adhesion between the deforming spheres. Adhesion at the contact points between soft particles *might* restrict the free diffusion of the particles, thereby inhibiting the stratification into two layers. pBA_25_ particles are solid elastic spheres, which will make only a point contact with other particles and be less likely to form strong adhesive contacts. They might be less likely to be restricted in stratification.

Once again, it appears as though having a uniform distribution of the small component (whether it is pBA_0_ or pBA_25_) worsens the adhesive properties of mixed films, whereas having a thin layer stratified near or on the top surface improves the adhesion.

## Conclusions

We have shown conclusively that stratification occurs upon the film formation of bimodal colloidal dispersions containing a mixture of small poly(butyl acrylate) and large poly(acrylate) particles, when Pe_S_ is sufficiently high. Tack adhesion properties are modified simply by changing the evaporation rate (via control of the temperature and relative humidity) to achieve a sufficiently fast evaporation of water. PSAs made from the same mixture of colloids had very different adhesive properties, depending on the film formation conditions.

As Pe_S_ was increased (at least above 19), enrichment of the small particles at the top surface was found with elastic recoil detection, to depths of 1 μm. Surface coverage or surface enrichment of small particles was shown with atomic force microscopy. Adhesive performance was improved, achieving a *W*_adh_ greater than that of the plain adhesive, observed in tack tests, as well as tunable failure strain, ϵ_failure_, arising from greater fibril extension with increasing Pe_S_.

Our results are in agreement with prior experimental work studying stratification in bimodal colloidal films^30,34^ and with investigations showing the benefits of bilayer systems with a gradient in properties,^13−17^ in particular the benefit of using a two-layered structure with a thin elastic surface.^13,17^ The stratification of the elastic pBA_25_ particles lead to a significantly higher plateau stress without sacrificing too much of the extension during fibrillation. As such, *W*_adh_ was greater than for the two individual components in the mixture.

Previous investigations achieved two layers via successive depositions, whereas in this present research we applied diffusiophoresis-driven stratification to design single deposition films with a gradient in properties, leading to improved adhesion properties. The strategy presented here could be extended to manufacture adhesives with a nontacky surface on top of an adhesive sublayer. Stratification of PSAs during the film deposition could lead to reduced production costs because of the shorter times and less energy required for manufacturing.

## Materials and Methods

### Materials

For the large component, we used a low-*T*_g_ commercial adhesive (named “PSA2” in this paper) derived from monomers of butyl acrylate and ethyl acrylate, synthesized by emulsion polymerization and received from Synthomer plc. For the small component, we used (non)cross-linked poly(butyl acrylate). Ethylene glycol dimethacrylate (EGDMA), sodium dodecyl sulfonate (SDS), potassium persulfate (KPS), acetone, isopropanol, and 1 M potassium hydroxide (KOH) were purchased from Sigma-Aldrich. Deuterated *n*-butyl acrylate (d9-nBA) was purchased from Polymer Source, Inc. All chemicals were used as received.

### Synthesis of Poly(butyl acrylate) Particles

Prior to each synthesis, a round-bottom flask was thoroughly cleaned by consecutively adding detergent, acetone, and isopropanol. Subsequently, the inner glass surface was etched by 0.1 M potassium hydroxide solution for 3 h and afterward flushed with ample Milli-Q water. Poly(nBA) colloids were synthesized according to a one-step emulsion polymerization. The particles called pBA_25_ were made with the addition of EGDMA as a covalent cross-linker at a concentration of 25 mol % calculated as *n*EGDMA/(*n*EGDMA + *n*nBA), where *n* is the number of moles. The particles called pBA_0_ contained no EGDMA.

For the nondeuterated, cross-linked latices (pBA_25_), 75 g of ingredients was mixed in a 25 mL round-bottom flask containing a 15 × 6 mm^2^ (*l* × *w*) oval Teflon-coated magnetic stirring bar. First, 6.3 mg of SDS was dissolved in 6.42 g of Milli-Q water by magnetic stirring to provide particle stabilization and fine-tuning of the size. Then, a total of 1.93 g of nBA and the cross-linker EGDMA were homogeneously mixed in a different vial and slowly added on top of the aqueous solution. Subsequently, the flask was sealed with a rubber septum. Oxygen was expelled from the reaction mixture by flushing and bubbling dry nitrogen for 15 min using a long injection needle, while stirring at 150 rpm, followed by 5 min at 60 °C. Finally, a solution of 158 mg of the thermally decomposing initiator KPS in 3.7 g of Milli-Q water was injected into the water phase using a long needle, after which the nitrogen inlet and outlet were removed. The reaction was continued for 20 h at 60 °C while stirring at 150 rpm. The final colloidal dispersions typically had a solids content of 20 wt %. The nondeuterated latexes were used for polymer characterization because the deuterium-labeled equivalents were produced in small volumes.

The synthesis protocol for the deuterated latices was identical except for the volumes due to the limited quantity of d9-nBA. Specifically, a 25 mL round-bottom flask was used, containing a 15 × 6 mm^2^ stirring bar, in which 6.3 mg of SDS and 6.42 g of Milli-Q water were mixed, and a total of 1.93 g of d9-nBA and EGDMA were added. Note that the higher density of *d*_9_-nBA compared to nondeuterated nBA caused the monomer to sediment to the bottom at room temperature, yet at 60 °C, its density was lower than that of the aqueous phase, causing migration to the top—an essential requirement for well-controlled emulsion polymerization.

### Particle Characterization

Particle sizes were determined via dynamic light scattering (DLS). Measurements of the diluted samples were performed on a Malvern Zetasizer Nanoseries (Nano S, ZEN1600), using a 4 mW 632.8 nm He–Ne red laser and an avalanche photodiode detector measuring light intensity at a detection angle of 173°. Glass transition temperatures were determined using differential scanning calorimetry (DSC) using a TA Instruments Discovery DSC 250 (Newcastle, DE). 75 μL of the wet samples (10 wt % solids) was drop-cast into pans and dried on a hot plate at 60 °C, such that the dry polymer (mass of 6–8 mg) was analyzed. A heat/cool/heat cycle was used, with a heating rate of 20 °C min^–1^ over a range from −80 to 80 °C. The *T*_g_ was determined from the second heating curve. The relative particle sizes presented in Figure 1 provide size ratios of α = 5.5 for the mixture with pBA_0_ small particles, and α = 6.5 for pBA_25_.

### Polymer Characterization

Molecular weights were determined by gel permeation chromatography (GPC). GPC analysis was performed on a Viscotek GPCMax VE 2001, which has three linear columns (7.5 × 300 mm^2^ PLgel mixed-D) operating at 35 °C and a flow rate of 1.0 mL/min with tetrahydrofuran (THF) as a mobile phase. PMMA standards were used to calibrate the GPC. Before injection, samples (2–4 mg/mL) were dissolved in THF overnight and filtered through 0.2 μm regenerated cellulose syringe filters.

To determine gel contents, 1 mm thick copolymer films with an initial weight of *W*_1_ were placed into cellulose extraction thimbles, using a Soxhlet extraction method in boiling THF for 24 h. The insoluble copolymer film was dried overnight in a vacuum oven at 40 °C and weighed (*W*_2_). The gel content, ϕ_gel_, was calculated as
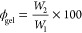
5The *z*-average values of the particle radius, *R*, are presented alongside values of the cross-linker concentration, molecular weight, *M*_w_, glass transition temperatures, *T*_g_, and size ratios, α, of the samples in Table 1.

### Particle Mixing and Film Formation

Mixed samples were stored on a shaker bench for 30 min prior to film casting. The colloidal mixtures were observed to remain stable, with no evidence for depletion flocculation. For IBA and AFM sample preparation, 400 μL was dropped onto 2 cm × 2 cm silicon wafers and spread uniformly on the substrate, yielding an initial wet film thickness, *H*, of 1 mm. For probe tack samples, 1500 μL was similarly drop-cast onto 2.5 cm × 7.5 cm glass slides, yielding 160 μm thick dry films. Prior to the film casting, the substrates were wiped with acetone and placed in a UV-ozone cleaner for 10 min to increase their hydrophilicity.

To produce a range of Pe_S_, samples were film formed under different conditions, detailed in Table 4. For film samples dried on the hot plate, the substrates were first allowed to equilibrate on the hot plate for 5 min.

**Table 4:** Summary of Film Formation Conditions and Corresponding Values of Pe_S_

environment	*T* (°C)	relative humidity (%)	*E*(nm/s)	Pe_S_ (pBA_25_)	Pe_S_ (pBA_0_)
hot plate	60	44	384	80	95
desiccator	20	15	80	19	22
desiccator	20	85	10	2	3

### Ion Beam Analysis

Films were analyzed at the Surrey Ion Beam Centre by performing elastic recoil detection, with a 2.6 MeV ^4^He^+^ beam incident on the surface at an angle of 75° to the sample normal. The beam had a diameter of approximately 1 mm. An 8 μm thick aluminum range foil was used to filter out any forward scattered ^4^He^+^ ions that may be incident on the ERD detector. A total charge of 10 μC of charged particles was collected from each sample. The detector geometry is shown in Figure S3 (Supporting Information). ERD spectra were analyzed and modeled using SIMNRA software,^61^ in which a simple (multi)slab model is employed to fit the data to a given film structure and produce a depth profile.^62^

To model the raw data, a single slab containing the approximate composition of a random 3:1 mixture of the two components, containing 33.3% C, 48.4% H, 13.3% O, and 5% D, is first used. When necessary to achieve agreement with the data, additional slabs with a different composition are added to the model. The D:H:C:O stoichiometry of the compositions of the slabs was set so that it corresponded to a mixture of deuterated pBA and PSA2. D and H were identified at the film surface through the corresponding energies of their peaks. Although the concentration of deuterium expected for a mixture containing large and small particle dispersions in a ratio of 3:1 is 10 at. %, during the analysis with SIMNRA, 5 at. % was found to be appropriate for all samples. This discrepancy could be due to uncertainty in the mixing process or deuterium losses from the film arising from beam-induced damage.

### Atomic Force Microscopy

Images were recorded on a Bruker Dimension Edge with Scan Asyst atomic force microscope, using Bruker’s Scan Asyst image optimization technique. This technique is a type of Peak Force Tapping that requires minimal user input for parameters, such as the set point, because they are automatically adjusted by a feedback loop to optimize the image, based on the information received about the sample surface. Height and adhesion maps are provided, in which height images provide topographic information, and adhesion images provide the relative tip–sample detachment force across the sample surface and is well described by Heinz and Hoh.^63^ For Scan Asyst imaging, a SCANASYST-AIR silicon tip on a silicon nitride cantilever was used, with a nominal resonant frequency of 70 kHz and a nominal spring constant of 0.4 N/m, as given by the manufacturer. Images were typically obtained using a scanning rate between 0.5 and 1 Hz.

### Probe Tack Adhesion

Probe tack adhesion measurements were performed on a testing rig (Texture Analyzer, TA-XT Plus, Stable Micro Systems, Godalming, UK) using a spherical polypropylene probe (1 in. diameter), a load force of 4.9 N, a test speed of 5 mm/s, and a contact time of 1 s.

During a tack test, a spherical probe is brought into contact with an adhesive film and then retracted from the film at a constant speed. The force required to withdraw the probe from the film is obtained as a function of distance and used to produce a stress–strain curve. Several useful parameters can be obtained from the stress–strain curves. By integrating the curve and multiplying by the dry film thickness, the total work of adhesion, *W*_adh_, is found. The maximum stress, σ_max_, is where the tensile load is at a maximum and represents the onset of cavity formation in the film. As the strain increases, these cavities continue to grow until σ_plateau_ at which point lateral growth stops, and continued deformation occurs by elongation of the cavity walls, known as fibrillation. σ_plateau_ is the stress required to stretch the fibrils, taken at the midpoint of the plateau strain. Film failure occurs either because of fibril detachment from the probe or the substrate, or by fracture within the fibrils, and the point at which this happens is defined as ϵ_failure_.

## Supporting Information Available

Rheology data for PSA2, pBA_0_, and their mixtures; fitted ERD spectra across the energy range; description of ERD experimental setup and energy calibration (PDF)
