# Supplementary material for: Diffusiophoresis-Driven Stratification in Pressure-Sensitive Adhesive Films from Bimodal Waterborne Colloids
Source: ACS Appl Polym Mater. 2023 Jan 25;5(2):1565–76. doi: 10.1021/acsapm.2c02044 (PMC9926484; doi:10.1021/acsapm.2c02044)
Supplement: Supplementary file 1 — ap2c02044_si_001.pdf [file ap2c02044_si_001.pdf]

Supporting Information

Diffusiophoresis-Driven Stratification in Pressure-Sensitive Adhesive Films from Bimodal Waterborne Colloids

Toby R. Palmer[1], Hanne M. van der Kooij[2], Rohani Abu Bakar[1], Callum D. McAleese[4], Mathis Duewel[3], Katja Greiner[3], Pierre Couture[4], Matthew K. Sharpe[4], Joseph L. Keddie[1]**

[1]Department of Physics, University of Surrey, Guildford, Surrey, GU2 7XH, UK

[2]Physical Chemistry and Soft Matter, Wageningen University, Wageningen, The Netherlands

[3]Synthomer Germany GmbH, Werrastraße 10, 45768, Marl, Germany

[4]Surrey Ion Beam Centre, University of Surrey, Guildford, Surrey, GU2 7XH, UK

**Corresponding Author: j.keddie@surrey.ac.uk*

S1. Rheology Data

Rheology data showing *G'*, *G''* and *tan(δ)/G'* as a function of angular frequency are presented in **Figure S1.**

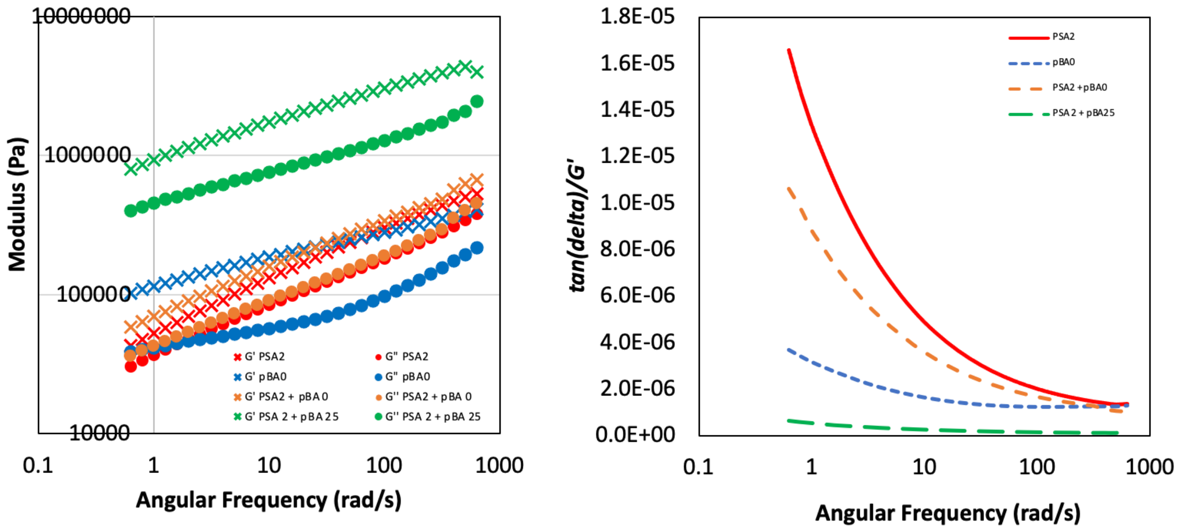

Figure S1. (a) *G′* and *G″* and (b) *tan(δ)/G′* from a frequency sweep at 25 °C.

S2. Original ERD Data with Best Fits

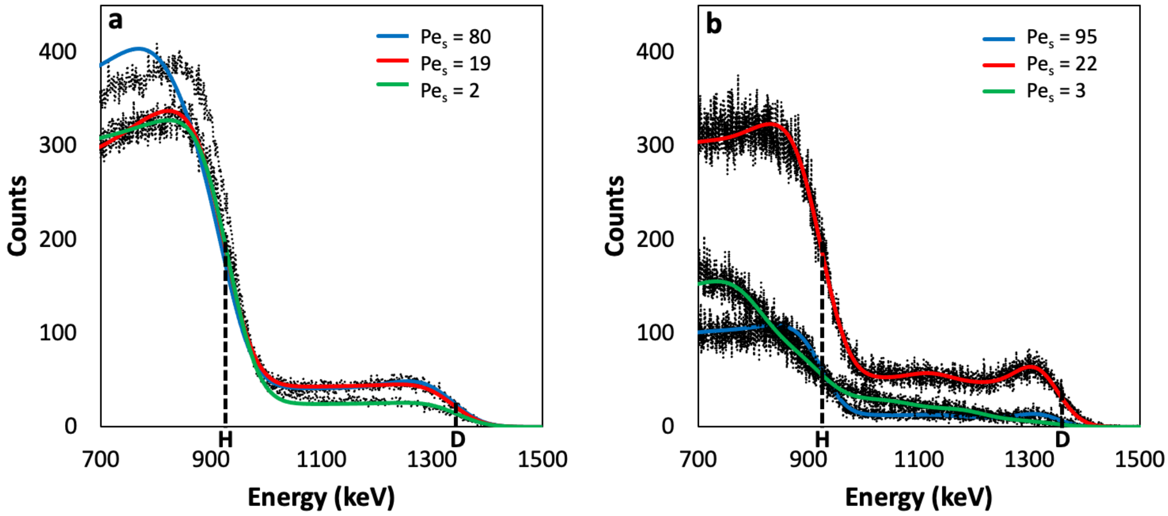

Figure S2. Original ERD spectra for films obtained with three different values of Pe_s from mixtures of PSA2 with (a) pBA_{25} and (b) pBA_0, without the normalization of the counts. The solid lines show the best fits to the model.

S3. ERD Energy Calibration & Fitting

The detector and beam set up is shown in **Figure S2**.

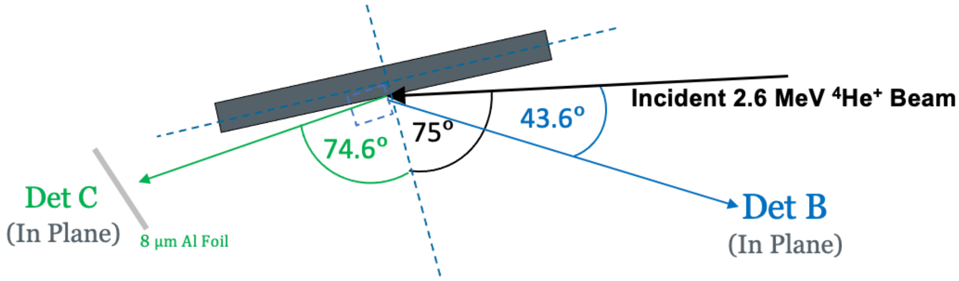

Figure S3. The detector and beam set-up for the ERD measurements. The beam is incident on the sample at an angle of 75° to the normal. Detector C was used for ERD. There is an 8 μm range foil in front of detector C, to prevent forward scattered He^+ ions.

The process for the energy calibration has been described in detail in a previous publication,[1] but the key energy calibration values used during the analysis in SIMNRA are included in **Table S1.**

Table S1. Calibration parameters used for ERD analysis.

	pBA_{25}	pBA_0
Energy per Channel (keV/Ch)	0.308	0.303
Calibration Offset (keV)	30	30
Detector Resolution (keV)	90	100
Solid Angle (mSr)	1.04	0.18

To model the raw data, we start by including a single slab containing the approximate composition of a random mixture of the two components, containing 33.3 % C, 48.4 % H, 13.3 % O and 5 % D. Where necessary, additional slabs can be added onto the surface that contain a different composition. This can be used to establish if any enrichment of the D containing small component has occurred. D and H can be identified based on the corresponding energy of the peaks. Although the concentration of deuterium expected for a mixture containing large and small particle dispersions in a ratio of 3:1 is 10\%, during the analysis with SIMNRA, 5 % was found to be appropriate for all samples. This could be due to uncertainty in the mixing process or deuterium losses from the film due to beam induced damage.

Reference

(1) Palmer, T.; van der Kooij, H.M.; Abu Bakar, R.; Duewel, M.; Greiner, K.; McAleese, C.D.; Couture, P.; Sharpe, M.K.; Smith, R.W.; Keddie, J.L. How Particle Deformability Influences the Surfactant Distribution in Colloidal Polymer Films. *Langmuir* **2022**, 38, 41, 12689-12701.
